# Assessment of choroidal vascularity index in juvenile idiopathic arthritis: implications for disease monitoring

**DOI:** 10.1007/s00417-025-06759-z

**Published:** 2025-02-01

**Authors:** İbrahim Edhem Yılmaz, Gizem Gürbostan Soysal, Veysel Doğru, Sevim Ayca Seyyar

**Affiliations:** 1 Ophthalmology Department, Gaziantep Islam Science and Technology University, Gaziantep, Turkey; 2 Gaziantep City Hospital Ophthalmology Clinic, Gaziantep, Turkey; 3 Gaziantep University Hospital Ophthalmology Department, Gaziantep, Turkey

**Keywords:** Juvenile Idiopathic Arthritis (JIA), Choroidal Vascularity Index (CVI), Optical Coherence Tomography (OCT), Uveitis, Pediatric Rheumatology

## Abstract

**Purpose:**

Juvenile Idiopathic Arthritis (JIA) can affect ocular structures, but choroidal involvement is not well understood. This study investigates the Choroidal Vascularity Index (CVI) in JIA patients compared to healthy controls and explores its relationship with disease activity.

**Methods:**

In this cross-sectional study, 35 JIA patients and 40 healthy controls underwent comprehensive ophthalmic examination and swept-source optical coherence tomography (SS-OCT). CVI, central macular thickness (CMT), and subfoveal choroidal thickness (SFCT) were measured. The Juvenile Arthritis Disease Activity Score (JADAS) was calculated for JIA patients. Statistical analysis included comparison between groups and correlation analysis.

**Results:**

JIA patients showed significantly lower CVI compared to controls (68.3 ± 2.5% vs. 72 ± 4.6%, *p* < 0.001). No significant difference was found in SFCT. CVI demonstrated a moderate negative correlation with JADAS (r = -0.368, *p* < 0.05). However, receiver operating characteristic (ROC) analysis revealed poor diagnostic performance of CVI for detecting JIA (AUC = 0.25).

**Conclusion:**

The study reveals reduced choroidal vascularity in JIA patients and a correlation between CVI and disease activity. While CVI shows limited diagnostic utility, it may serve as a potential marker for monitoring inflammatory burden and treatment response in JIA. Further research is needed to establish its clinical utility fully.

**Supplementary Information:**

The online version contains supplementary material available at 10.1007/s00417-025-06759-z.

## Key Messages

*What is known*

- Juvenile Idiopathic Artritis (JIA) can affect ocular structures, with uveitis being a well-recognized complication.

*What is new*

- Choroidal Vascularity Index (CVI) unveils hidden vascular changes in JIA patients, even in the absence of clinically apparent uveitis.
- CVI exhibits an inverse relationship with JIA disease activity, potentially serving as a "window" into systemic inflammation.
- While not a standalone diagnostic tool, CVI emerges as a promising non-invasive biomarker for monitoring JIA progression and treatment efficacy.

**Supplementary Information:**

The online version contains supplementary material available at 10.1007/s00417-025-06759-z.

## Introduction

Juvenile idiopathic arthritis (JIA) is the most prevalent chronic rheumatic disease affecting the paediatric population, occurring in about 1 in 1,000 children worldwide [1]. JIA is considered a disease characterised by the persistence of arthritis for at least six weeks in children under 16 years of age, though it represents a broad spectrum of illnesses with different clinical phenotypes [2]. While the aetiology remains unclear, JIA is thought to arise from a complex interplay among genetic predisposition and an increasing plethora of environmental triggers causing immune dysregulation [3].

Besides the musculoskeletal system, JIA is often associated with the involvement of other organs, most notably the eyes. The most common extra-articular manifestation is uveitis, occurring in as high as 30% of all JIA patients, with a potentially insidious course without symptoms and significant risk of severe visual impairment [4, 5]. This emphasizes the need for early diagnosis and appropriate management of ocular involvement in JIA.

Essentially, the diagnosis of JIA is based on clinical examination, supported by laboratory findings and imaging studies. The classification criteria developed by International League of Associations for Rheumatology (ILAR) remain the gold standard today for diagnosis [6]. These criteria do not identify subclinical inflammation or predict disease outcomes. Ophthalmic screening can be done periodically but early ocular changes—especially in uveitis related to JIA—can only be discovered when they already cause irreversible damage [7].

Timely diagnosis and intervention remain critical to improving outcomes in JIA. Early treatment minimizes joint damage, growth disturbances, and much vision loss [8]. Recent advances encourage early, aggressive treatment approaches in a 'window of opportunity' to achieve better disease control and even induce remission [9]. However, deficiencies in current diagnostic feeder systems have taken into account the detection of early ocular and systemic changes and have raised interest in novel biomarkers, including imaging-based metrics.

Equally varied, one of the promising areas is the examination of the choroid using enhanced depth imaging optical coherence tomography (EDI-OCT) and swept-source OCT (SS-OCT)—one of the most vascularized layers of the eye [10, 11]. Advanced imaging methods enable the investigation of the choroid as a well-detailed structure, allowing for insights into ocular and systemic vascular changes. Thus, it is an upcoming novel marker of vascular health—a quantified ratio of the luminal area to total choroidal area. It was studied in various diseases like diabetic retinopathy, age-related macular degeneration, and Behçet's disease, indicating its potential as a stable quantitative indicator of choroidal vascularity.

These changes are of particular interest given the critical role that the choroid might play in the pathogenesis of JIA-associated uveitis. Changes in choroidal thickness have indeed been described among JIA patients without active uveitis, but changes in thickness alone may not accurately reflect changes in the underlying vasculature [12]. Choroidal vascular index (CVI) is thus a more sensitive indicator of choroidal health, given that age, refractive error, and even diurnal variations exert less influence on it, and could more accurately reflect microvascular changes due to inflammation associated with JIA [13–15].

Systemic inflammation in JIA seems to be associated with vascular dysfunction and accelerated atherosclerosis, and it may also be expressed in the choroid [16]. Thus, CVI could represent a non-invasive marker of ocular and systemic inflammation in JIA. Despite such possibilities, the relationship between CVI and JIA is still under-explored; only a few studies have looked into the correlations of CVI with clinical markers of activity of the JIA.

This will be one of the first studies that investigates CVI and other ocular parameters in JIA patients in comparison with healthy controls, attempting to fill this knowledge gap. We also analyse the correlation between CVI and generally accepted JIA disease activity measures, such as the Juvenile Arthritis Disease Activity Score, JADAS, in order to conclude if CVI can be used as a good indicator of disease activity and ocular involvement [17]. In reviewing the CVI in relationship to other OCT-derived measures, we will further elucidate ocular manifestations of JIA and further its validity as an early marker for subclinical inflammation to enhance the management of the disease and improve long-term outcomes in JIA patients.

## Methods

### Study design and participants

The study was a cross-sectional retrospective study conducted at a University Hospital, following the Declaration of Helsinki and approved by the Institutional Review Board. Written informed consent was obtained from all participants.

The study involved two groups: JIA Group, patients aged 4–16 with confirmed JIA diagnosis, and Control Group, age- and sex-matched healthy children without systemic or ocular diseases, based on ILAR criteria.

The study excluded individuals with a history of ocular surgery or trauma, refractive errors exceeding ± 6.00 dioptres spherical equivalent, media opacities preventing clear OCT imaging, and inability to cooperate with OCT examination.

### Data collection

#### Clinical assessment

Participants underwent a comprehensive ophthalmic examination, including best-corrected visual acuity (BCVA), refraction, biomicroscopy, dilated fundus, and intraocular pressure (IOP) measurement. For JIA patients, additional data was collected, including subtype, disease duration, current medications, and JADAS.

#### OCT imaging and analysis

OCT examinations were performed using a swept-source OCT device (Heidelberg Engineering, Heidelberg, Germany) with a central wavelength of 1050 nm, an acquisition speed of 100,000 A-scans per second, and axial and transverse resolutions of 8 μm and 20 μm in tissue, respectively.

The following scans were obtained for each eye:

1. Macular cube scan (7 × 7 mm area centred on the fovea, 512 A-scans × 256 B-scans)
2. Radial scan pattern (12 radial B-scans centred on the fovea, 1024 A-scans each)
3. Enhanced Depth Imaging (EDI) mode for choroidal imaging

All scans were performed between 9:00 AM and 11:00 AM to minimize diurnal variations. Only high-quality images with a signal strength index > 60 were included in the analysis.

#### Measurement protocols

The following parameters were measured:

1. *Central Corneal Thickness (CCT):* Measured using the built-in CCT map from the anterior segment module of the OCT device.
2. *Central Macular Thickness (CMT):* Automatically calculated as the average thickness in the central 1 mm diameter of the ETDRS grid on the macular thickness map.
3. *Subfoveal Choroidal Thickness (SFCT):* Measured manually using the built-in caliper tool, from the outer border of the retinal pigment epithelium to the choroid-sclera interface at the foveal center.
4. *Choroidal Vascularity Index (CVI):* Calculated using a validated semi-automated image binarization technique:
  - A 1500 μm wide area of the subfoveal choroid was selected from the EDI-OCT image.
  - The image was binarized using ImageJ software (version 1.52a, National Institutes of Health, USA) with Niblack's auto local threshold tool.
  - The luminal area and total choroidal area were calculated.
  - CVI was derived as the ratio of luminal area to total choroidal area, expressed as a percentage.

All OCT measurements were performed by two independent, masked graders. In cases of discrepancy > 10%, a third grader adjudicated the measurement. The average of the two closest measurements was used for analysis.

The study ensured the reliability of measurements by performing all OCT scans by a single experienced operator, calibrating the device daily, and assessing intra-grader and inter-grader reliability using intraclass correlation coefficients.

#### Statistical analysis

Statistical analyses were conducted using SPSS software (version 26.0, IBM Corp., Armonk, NY, USA). The normality of data distribution was assessed using the Shapiro–Wilk test. Continuous variables were expressed as mean ± standard deviation or median (interquartile range) as appropriate. Categorical variables were presented as frequencies and percentages.

The study compared the JIA and control groups using independent t-tests or Mann–Whitney U tests for continuous variables and Chi-square tests for categorical variables.

Correlations between CVI and other parameters (age, CCT, IOP, CMT, SFCT, JADAS) were evaluated using Spearman's correlation coefficients.

Receiver operating characteristic (ROC) curve analysis was conducted to assess the diagnostic performance of CVI for detecting JIA-associated ocular changes. For all analyses, a two-tailed p-value < 0.05 was considered statistically significant.

## Results

A total of 75 participants were included in the study: 35 patients with Juvenile Idiopathic Arthritis (JIA) and 40 healthy controls. The demographic and clinical characteristics of the study population are summarized in Table 1.

**Table 1:** Demographic and Clinical Characteristics of Study Participants

Characteristic	JIA Group (*n* = 35) (mean ± SD)	Control Group(*n* = 40) (mean ± SD)	P-value
Age (years)	10.6 ± 4.2	10.5 ± 3.8	0.91(Mann–Whitney U test)
Sex (Female/Male)	21/14	23/17	0.85(Chi-square test)
Refraction (SE-Diopters)^α^	0(−0.175—0.25)	0(−0.5—0.25)	0.45 (Mann–Whitney U test)
***JIA Subtype, n (%)***			
Oligoarticular	16 (45.7%)	-	-
Polyarticular	10 (28.5%)	-	-
Systemic	3 (8.5%)	-	-
Enthesitis-related	6 (17.1.6%)	-	-
Disease Duration (months)^β^	24 (4–28)**	-	-
JADAS	12.33 ± 11.2	-	-

^**α**^Data presented as median (interquartile range); ^**β**^: Data presented as mean (interquartile range) JIA: Juvenile Idiopathic Arthritis, JADAS: Juvenile Arthritis Disease Activity Score

There were no significant differences in age or sex distribution between the JIA and control groups. Among JIA patients, oligoarticular JIA remained the most common subtype, followed by polyarticular JIA.

JIA patients showed significantly lower CVI compared to healthy controls. There were no significant differences in IOP, CCT, or SFCT between the groups (Table 2).

**Table 2:** Comparison of Ocular Parameters between JIA and Control Groups

Measurement	JIA Group (*n* = 35)	Control Group(*n* = 40)	*P*-value
IOP (mmHg)	14.11 ± 3.7	13.25 ± 3.6	0.283
CCT (μm)	535 ± 30	523 ± 26	0.059
CMT (μm)	240 ± 25.7	230 ± 17.5	0.04*
SFCT (μm)	343 ± 61	334 ± 58	0.258
CVI (%)	68.3 ± 2.5	72 ± 4.6	< 0.001**

*IOP* Intraocular Pressure, *CCT* Central Corneal Thickness, *CMT* Central Macular Thickness, *SFCT* Subfoveal Choroidal Thickness, *CVI* Choroidal Vascularity Index, *: Independent t-test; **: Mann–Whitney U test

**Fig. 1:**
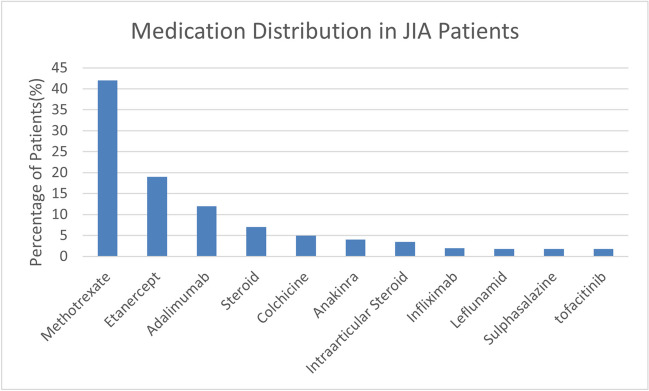
Distribution of Medications in JIA Patients The bar chart illustrates the percentage distribution of various medications used by Juvenile Idiopathic Arthritis (JIA) patients in the study population. Methotrexate was the most commonly prescribed medication, with nearly 70% of patients receiving it. Etanercept and Adalimumab were the second and third most common treatments, prescribed to approximately 30% and 20% of patients, respectively. Other medications, including steroids, colchicine, and intra-articular steroids, were used less frequently. Medications such as anakinra, leflunomide, tofacitinib, sulfasalazine, and infliximab were prescribed to a small percentage of patients

Figure 1 illustrates the distribution of medications in JIA patients. The analysis of medication distribution among JIA patients revealed that methotrexate was the most commonly prescribed drug, utilized in approximately 42% of cases. Biologic agents, particularly etanercept and adalimumab, were also frequently employed, with etanercept being the second most prevalent medication (19% of patients), followed by adalimumab (12%), indicating a significant role for targeted immunomodulatory therapy in managing JIA.

**Fig. 2:**
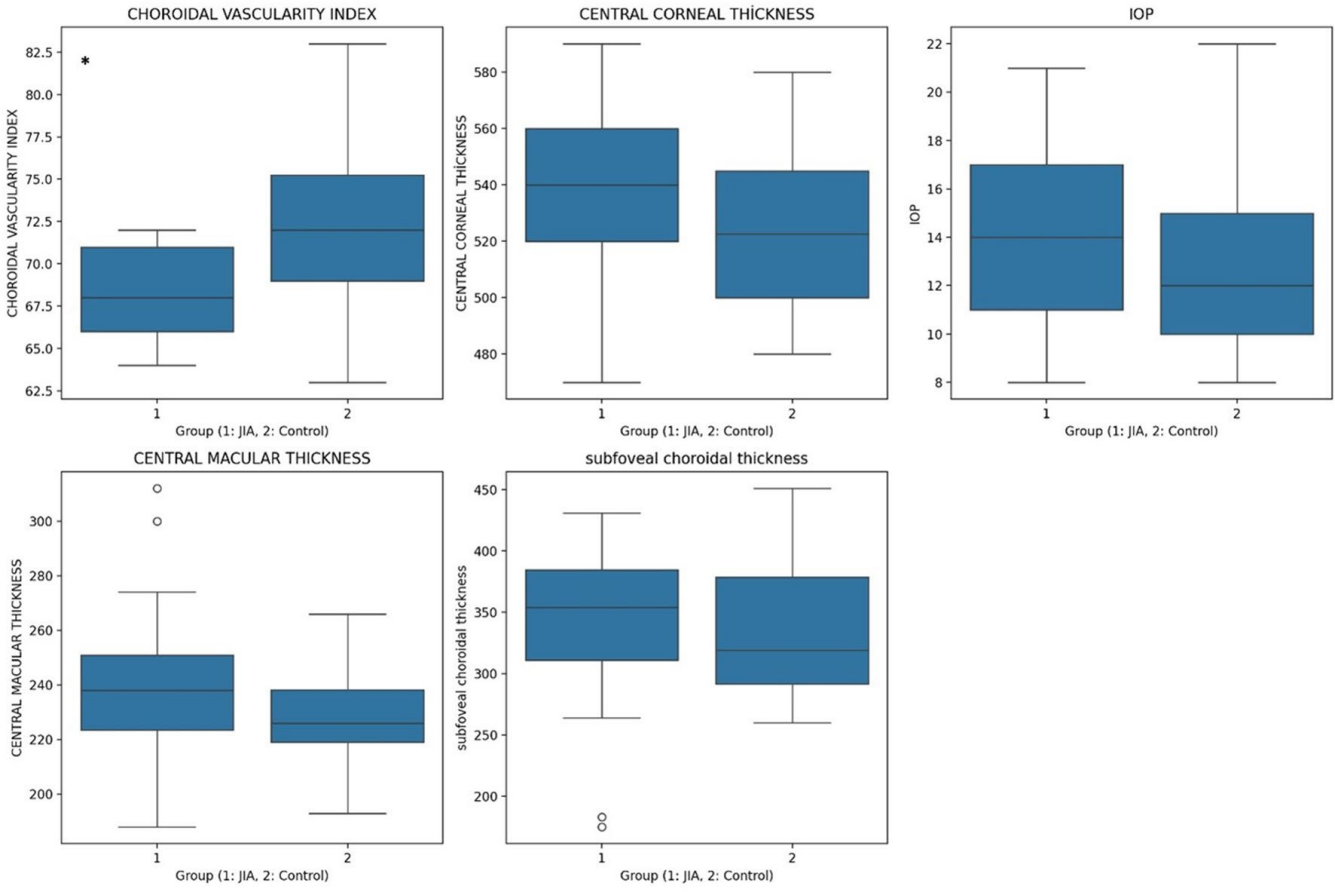
Comparison of Ocular Parameters Between JIA Patients and Healthy Controls. The box plots represent the comparison of various ocular parameters between Juvenile Idiopathic Arthritis (JIA) patients (Group 1) and healthy controls (Group 2). The parameters include Choroidal Vascularity Index (CVI), Central Corneal Thickness (CCT), Intraocular Pressure (IOP), Central Macular Thickness (CMT), and Subfoveal Choroidal Thickness (SFCT). ( *: 0.05 < *p*)—CVI: JIA patients exhibit significantly lower CVI compared to controls, suggesting reduced choroidal vascularity in the JIA group.—CCT: There is no significant difference in central corneal thickness between JIA and control groups.—IOP: Intraocular pressure is slightly elevated in the JIA group compared to controls.—CMT: Central macular thickness shows a small increase in JIA patients, although not significantly different from controls.—SFCT: While subfoveal choroidal thickness (SFCT) is higher in JIA patients, the difference is not statistically significant

Figure 2 illustrates the distribution of CVI and other measurement values in JIA patients and healthy controls.

The results show a weak positive correlation between IOP and central corneal thickness (0.39), and a weak negative correlation between CVI and JADAS, which are statistically significant (−0.368) (Table 3 ).

**Table 3:** Correlation matrix of measurements in JIA patients

	CVI	Age	CCT	IOP	CMT	SFCT	JADA**S**	Disease Duration
CVI	1	0.237	−0.235	−0.169	−0.057	0.036	−0.368*	0.045
Age	0.237	1	−0.044	0.105	0.093	−0.002	−0.07	0.264
CCT	−0.235	−0.044	1	0.396	0.084	0.197	0.071	−0.205
IOP	−0.169	0.105	0.4944*	1	−0.038	−0.179	0.06	−0.132
CMT	−0.057	0.093	0.084	−0.038	1	0.077	0.208	−0.208
SFCT	0.036	−0.002	0.197	−0.179	0.077	1	0.061	0.114
JADAS	−0.368	−0.077	0.071	0.068	0.208	0.061	1	−0.225
Disease Duration	0.045	0.264	−0.205	−0.132	−0.208	0.114	−0.225	1

* *p* < 0.05, Spearman's Rank correlation coefficient

**Fig. 3:**
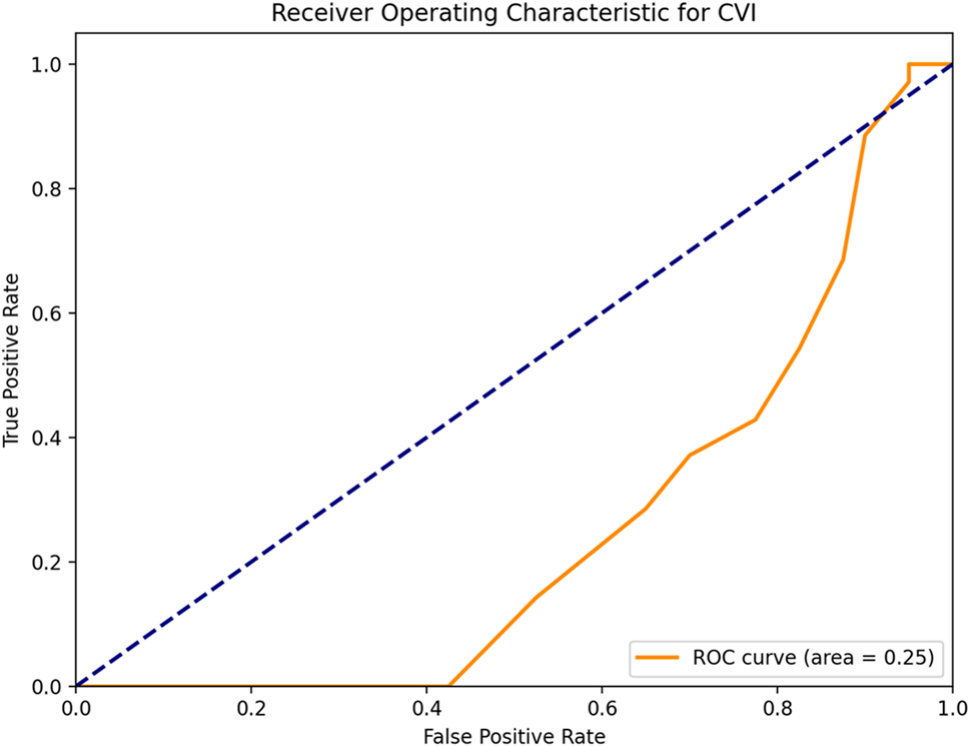
ROC Curve for CVI In Distinguishing JIA Patients from Healthy Controls*.* The Receiver Operating Characteristic (ROC) curve illustrates the diagnostic performance of the Choroidal Vascularity Index (CVI) in differentiating Juvenile Idiopathic Arthritis (JIA) patients from healthy controls. The orange curve represents the ROC plot, and the dashed diagonal line denotes a random classifier (area under the curve [AUC] = 0.5). The calculated area under the curve (AUC) for CVI is 0.25, indicating poor discriminatory power for CVI as a diagnostic marker in this sample. An optimal threshold of 64.00 yields high sensitivity but very low specificity, suggesting that while CVI can correctly identify most JIA cases, it also results in a high number of false positives

The ROC analysis was successfully completed after converting the group labels to binary values. The Area Under the Curve (AUC) is quite low, indicating poor diagnostic performance of CVI for detecting JIA. The low AUC of 0.25 indicates that the test performs worse than random chance. The optimal threshold of 64.00 yields high sensitivity but very low specificity at the optimal threshold, suggesting that while CVI can identify most JIA cases, it also results in many false positives (Fig. 3).

## Discussion

This study reveals a significant reduction in CVI among JIA patients compared to healthy controls, highlighting that JIA affects the posterior segment of the eye, particularly the choroidal vasculature. These results expand the understanding of JIA’s ocular manifestations beyond anterior segment involvement, suggesting that systemic inflammation in JIA influences not only the anterior but also the posterior ocular structures [18, 19].

The lower CVI in JIA patients despite higher SFCT reflects the complexity of inflammatory processes in JIA. While SFCT captures overall choroidal thickness, which can increase due to inflammatory stromal edema, CVI specifically reflects the vascular component. Thus, although the stromal component may expand with inflammation, vascular remodeling or damage from chronic inflammation can reduce the vascular area, leading to a lower CVI. This underscores the idea that inflammatory diseases like JIA cause vascular changes that may not always correlate directly with structural thickness [12]. These choroidal vascular changes may be initiated by immune-mediated endothelial damage and microvascular compromise, contributing to CVI reduction despite preservation or increase of overall choroidal thickness [14].

A moderate negative correlation between CVI and JADAS further substantiates the influence of systemic inflammation on choroidal vascular alterations. The inverse relationship suggests that increased disease activity correlates with decreased choroidal vascularity, positioning CVI as a potential marker for evaluating inflammatory burden in JIA patients. This finding supports the notion that increased inflammatory activity results in enhanced vascular damage within the choroid. Cytokine-mediated inflammation, particularly through TNF-alpha and IL-6, contributes to endothelial dysfunction, hypoxia, and vascular remodelling, ultimately leading to diminished cardiovascular integrity as disease activity increases [20].

Although CVI showed a significant correlation with JADAS, it demonstrated weaker or non-significant correlations with other ocular parameters, such as SFCT and CMT. This finding highlights the complexity of ocular involvement in JIA, indicating that CVI may offer unique insights into vascular changes that are independent of structural modifications in the choroid. Despite the increase in SFCT, the vascular component assessed by CVI exhibited a decrease, indicating the complex influence of inflammation on ocular tissues. Balaskas et al. [21] highlighted that anterior chamber inflammation can induce subclinical macular oedema, reflecting a dynamic inflammatory cascade involving the posterior segment. This interplay between anterior and posterior segment inflammation in JIA underscores the multifaceted ocular involvement in the disease. While the correlation between CMT and systemic inflammation in our cohort was weak, these findings emphasize the complexity of retinal changes in JIA and warrant further investigation into the inflammatory mechanisms at play.

Our findings align with existing research on the ocular complications of JIA, particularly the role of posterior segment involvement [14, 18, 22]. While much of the prior literature, including studies by Heiligenhaus et al. [5] and Gueudry et al. [23], has focused on anterior uveitis as the primary manifestation of JIA-related eye disease, our study broadens this perspective by highlighting choroidal involvement. The reduction in CVI observed in this study complements earlier work that reported choroidal changes in JIA patients, such as the study by Yılmaz Tuğan et al. [18], even in cases without clinical uveitis. By quantifying choroidal vascularity, our study provides a more precise assessment of posterior segment alterations, which may be more closely tied to systemic inflammatory status than previously understood.

The distribution of medications among JIA patients in our study reflects current treatment strategies, with methotrexate (42%) and biologic agents such as etanercept (19%) and adalimumab (12%) being the most commonly prescribed. The systemic administration of steroids or immunosuppressants may significantly influence CVI, as these therapies modulate vascular structures and reduce inflammation. None of our patients were on topical corticosteroids, and no cases of uveitis were recorded, minimizing these factors as potential confounders. Previous studies, such as those by Kotaniemi et al. [24], have noted that biologics can reduce ocular complications in JIA, but their specific effects on the posterior segment remain underexplored. The immunomodulatory actions of these medications could influence vascular structures like the choroid and partly explain the diagnostic challenges reflected in the ROC curve analysis. Future longitudinal studies are needed to determine whether CVI changes are primarily driven by these therapies or intrinsic disease mechanisms [25–27].

Although the CVI demonstrated a significant difference between JIA patients and healthy controls, the ROC analysis indicated poor standalone diagnostic performance (AUC = 0.25). This suggests that while CVI alone may not be effective in distinguishing JIA from healthy individuals, its clinical utility could be enhanced when combined with other biomarkers or imaging parameters. Low specificity at the optimal threshold would suggest that CVI is more sensitive to early changes in the choroid and could therefore give false positives if used in isolation.

The association of CVI with JADAS nonetheless supports the use of CVI as a "monitoring tool" rather than a sole diagnostic marker. Specifically, the incorporation of CVI into routine ophthalmic assessments may provide better monitoring of disease progression or therapeutic response in patients with JIA, where systemic inflammatory markers do not always incompletely represent localized ocular inflammation. Unlike more conventional inflammatory markers, CVI may provide unique insights into how systemic inflammation affects the eye over time [28] With the fact that clinicians measure CVI regularly, early detection of choroidal involvement may be possible, thereby interceding before overt clinical symptoms of uveitis.

If standard systemic markers are not sensitive or adequate to capture subclinical ocular changes, CVI might fill this need as a non-invasive means of quantification in ocular health. This may be especially pertinent for patients undergoing biologic or other immunomodulatory treatments, as the supplementary data from CVI could signify the efficacy of these therapies and guide the modification of treatment protocols [28, 29].

This study has several potential limitations, including a small sample size, a cross-sectional design, and insufficient control for confounding factors such as medication usage and disease duration. Larger multicentre studies are needed to confirm these findings and further investigate possible differences among the various JIA subtypes. Longitudinal studies that follow the course of CVI over time and with treatment are needed; this would further elucidate how choroidal vascularity changes with disease activity.

## Conclusion

The study shows that CVI decreases significantly among JIA patients, supporting the effect of systemic inflammation on choroid. While CVI is of limited diagnostic utility, the observation that it correlates with disease activity suggests that this measurement may be a useful tool to follow inflammatory burden and treatment response. Further research is needed to fully establish its clinical utility.

## Supplementary Information

Below is the link to the electronic supplementary material.

Supplementary file1 (XLSX 18 KB)
